# Variation in the thermal and dehydration regime below Central America: Insights for the seismogenic plate interface

**DOI:** 10.1016/j.isci.2023.107936

**Published:** 2023-09-17

**Authors:** Rui Qu, Yingfeng Ji, Lijun Liu, Weiling Zhu, Ye Zhu, Chaodi Xie, Shoichi Yoshioka, Haris Faheem, Lin Ding

**Affiliations:** 1 State Key Laboratory of Tibetan Plateau Earth System, Environment and Resources (TPESER), Institute of Tibetan Plateau Research, Chinese Academy of Sciences, Beijing 100101, China; 2 University of Chinese Academy of Sciences, Beijing 100049, China; 3 State Key Laboratory of Lithospheric Evolution, Institute of Geology and Geophysics, Chinese Academy of Sciences, Beijing 100029, China; 4 University of Illinois at Urbana-Champaign, Urbana, IL, USA; 5 Geophysics Department, School of Earth Sciences, Yunnan University, Kunming 650500, China; 6 Research Center for Urban Safety and Security, Kobe University, Kobe, Japan; 7 Department of Planetology, Graduate School of Science, Kobe University, Kobe, Japan

**Keywords:** Earth sciences, Geology, Tectonics, Geophysics

## Abstract

Slow earthquakes predominant in Costa Rica indicate unstable faulting of segmented Central American megathrusts, but the recurrence of episodic tremors and slips reported to precede a giant earthquake remains still enigmatic. The underlying mechanism is related to the variation in the coupling along the heterogeneous subduction interface which is poorly understood. In this study, we used up-to-date 3D thermal modeling to provide insights into the along-strike variation in the thermal state and hydraulic distribution beneath the Central American subduction zone. Our results show that the subducted Cocos Plate is much warmer than previously estimated, and the slab geometry exhibits remarkable perturbations along the trench. We found that the regions of large dehydration rate along the slab are consistent with the seismicity occurrence depth beneath the Moho. Below the Nicoya Peninsula and the Guatemala-Nicaragua segment of megathrusts, fluids derived from subducted slab result in increased pore fluid pressures and subsequent recurrence of slow slip events and regular earthquakes.

## Highlights

• Cold mantle wedge (<500°C) is constrained at depths of 40∼60 km below Central America
• Surface temperature of subducted Cocos Plate is warmer than previously estimated
• Recurrence of fast-slow earthquakes is facilitated by downdip slab dehydration
• Slow earthquakes likely occur offshore Guatemala-Nicaragua warm segments

## Graphical abstract

**Figure:**
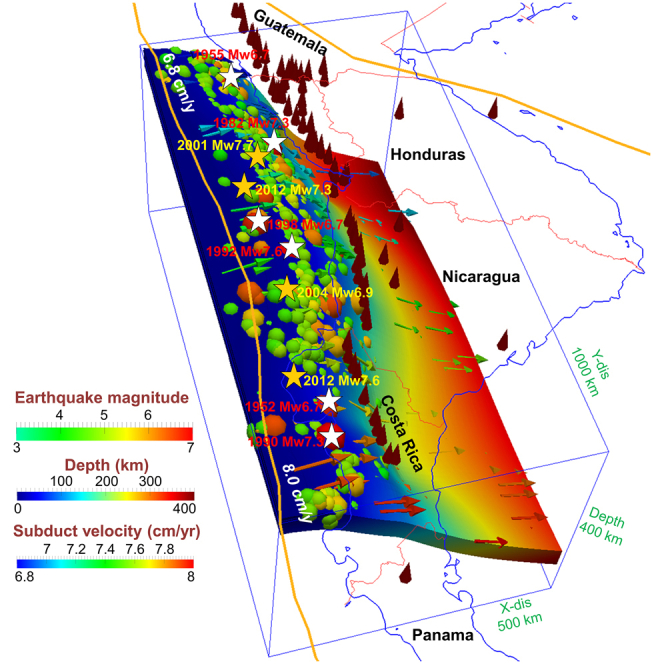

## Introduction

**Figure 1:**
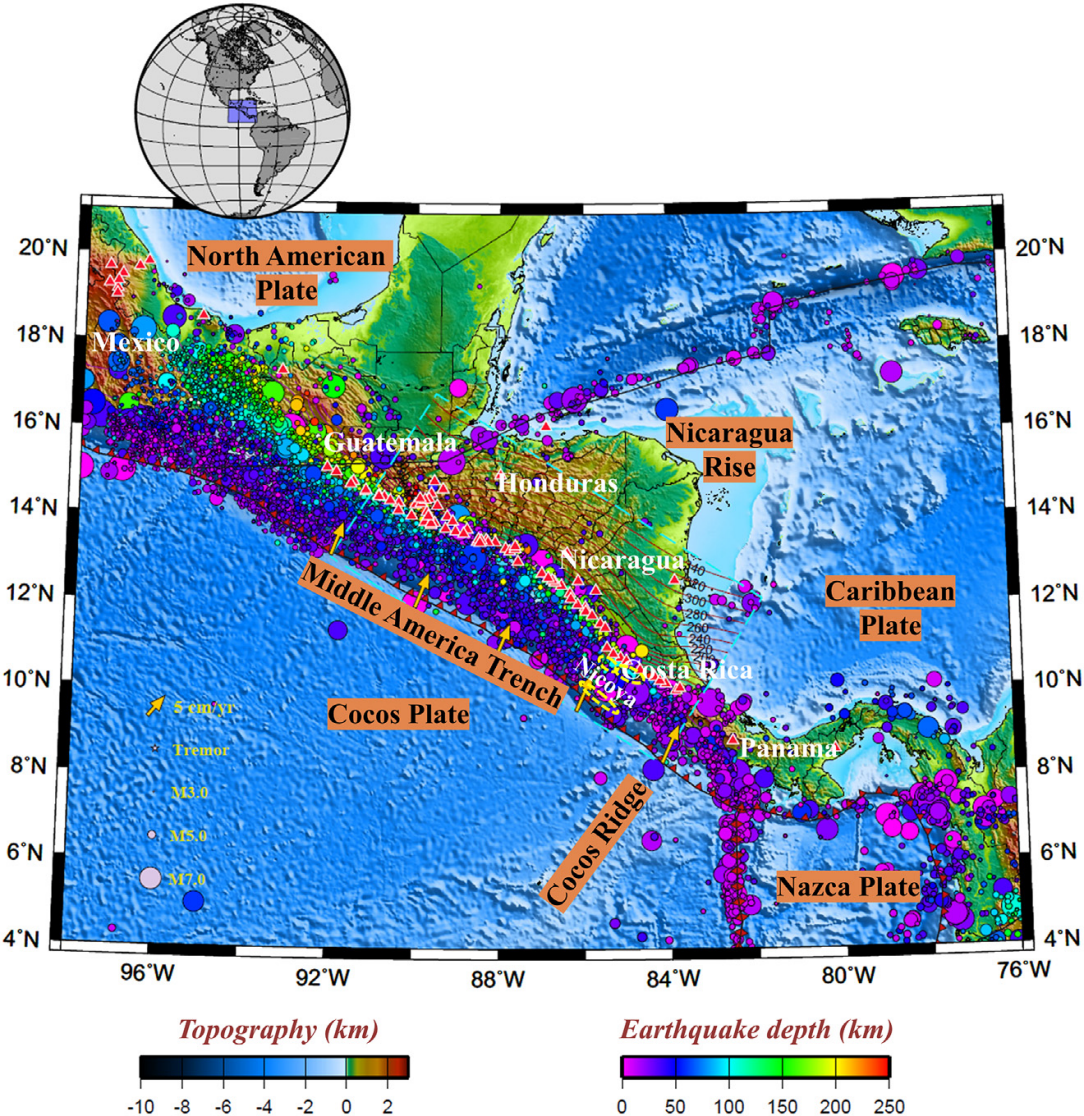
Tectonic map of Central America The background color indicates the surface topography (ETOPO^4^). The cyan dashed box delineates the study region. The yellow arrows denote the direction (orientation) and velocity (length) of subduction, and the red barbed lines mark convergent plate boundaries (with the teeth on the overriding plate). The red curves represent the isodepth contours of the subducted Pacific plate with a contour interval of 20 km (Slab2.0^5^). Red triangles indicate arc volcanoes.^3^ The solid circles indicate the epicenters of all earthquakes from January 2000 to December 2010 (IRIS^6^) and M > 5.5 earthquakes from January 1900 to December 2000 (Centennial^7^). Earthquake depths are indicated by the circle colors. The yellow ellipses with dashed lines represent the distribution of slow earthquakes below the Nicoya Peninsula, including the low-frequency earthquakes (LFEs^8^), large slip areas of the 2007 slow slip event (SSE^9^), and very-low-frequency earthquakes (VLFEs^10^).

Much of Central America is located at the westernmost margin of the Caribbean Plate, which overrides the slab of the Cocos Plate subducting along the Middle America Trench (Figure 1). The Cocos Plate converges obliquely toward the Caribbean Plate at a rate that increases from ∼6.8 cm/year offshore southern Guatemala to ∼8.0 cm/year offshore central Costa Rica.^1^ As a consequence of this rapid convergence, the Central American subduction zone is one of the most volcanically and seismically active regions on Earth. Approximately 75 basaltic to dacitic volcanoes are distributed principally along a line that closely parallels the Caribbean Plate boundary, forming a 1,500 km long volcanic arc that extends from southwestern Guatemala to central Panama (e.g., Leeman et al. and Siebert et al.^2^^,^^3^).

**Figure 2:**
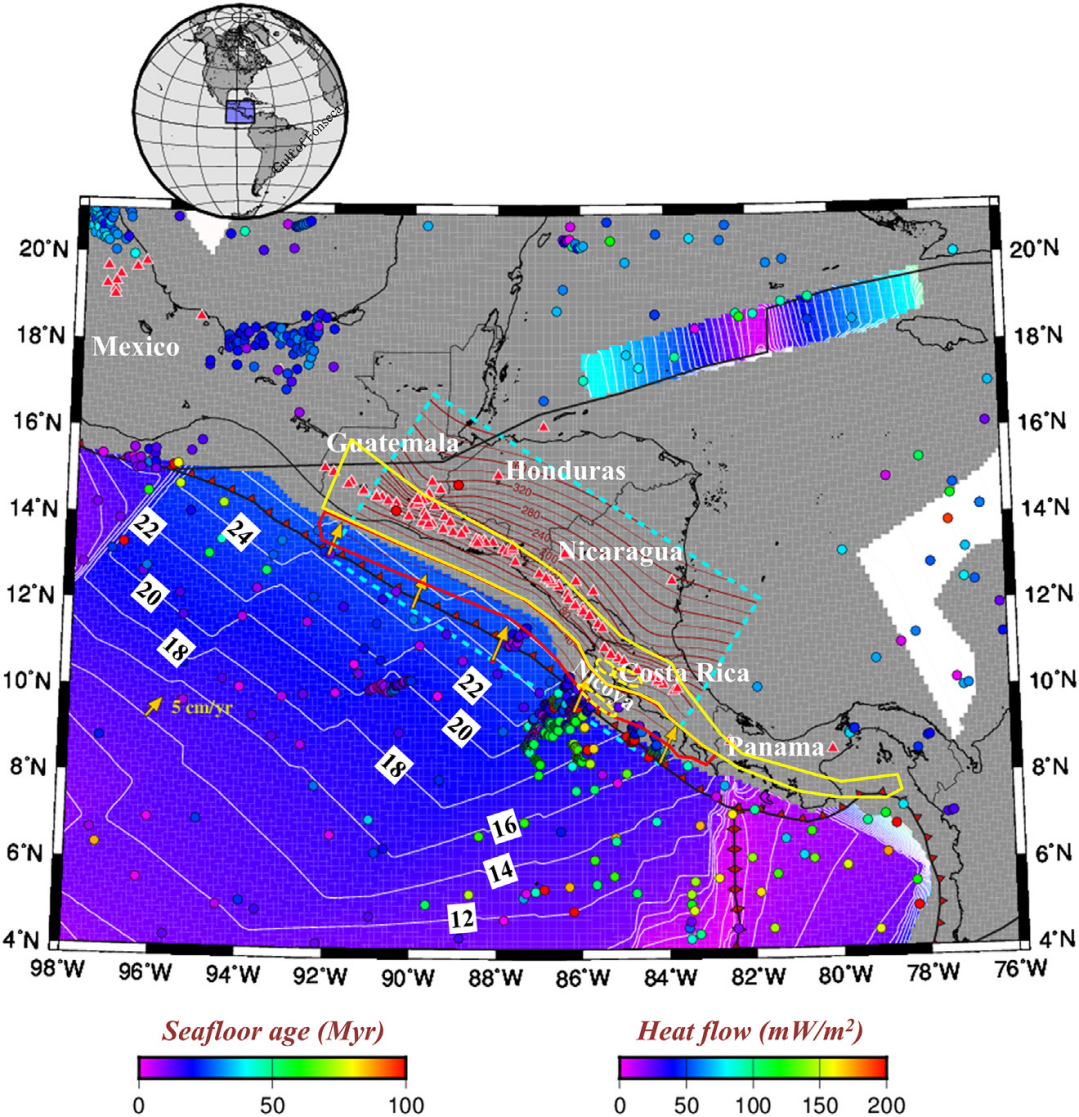
Seafloor age distribution and heat flow observations in Central America Solid circles represent the observations from the Global Heat Flow Database.^16^ The seafloor ages are derived from EarthByte.^17^ Red triangles indicate arc volcanoes.^3^ The yellow dashed lines represent the slow earthquakes below the Nicoya Peninsula.^18^ Red and yellow polygons indicate the interplate seismogenic zone (10–40 km depth) and intraplate seismogenic zone (>40 km depth), respectively.^14^

**Figure 3:**
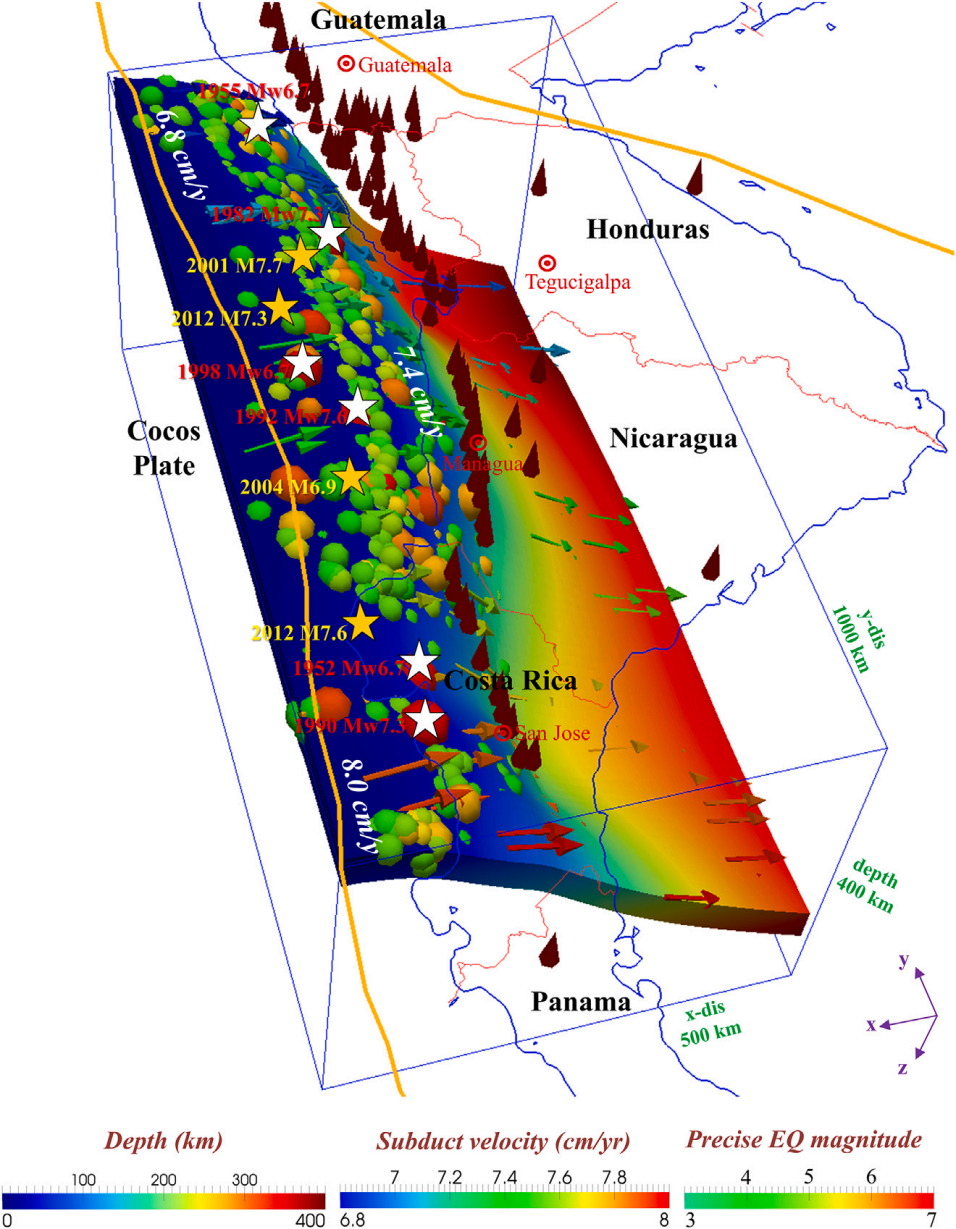
Model parameters and boundary conditions The seismic events are plotted as colored spheres on the plate interface, with the color indicating the earthquake magnitude. Red cones indicate active arc volcanoes.^3^ Arrows indicate the direction (orientation) and velocity (color) of plate subduction (MORVEL,^1^). White and yellow stars indicate the M > 5.5 earthquakes in the past century (1900–2000, Centennial^7^) and current century (2000–2014^19^), respectively.

Central America is underlain by a clearly defined Wadati-Benioff zone, whose maximum depth decreases southeastward, from ∼250 km beneath Nicaragua to ∼125 km beneath central Costa Rica (e.g.,^11^^,^^12^^,^^13^). Alvarado et al.^14^ assimilated all seismically active regions of the Central American subduction zone and proposed a new regional seismic hazard zonation comprising both an interplate seismogenic zone (10–40 km depth) and an intraplate seismogenic zone (>40 km depth) (Figure 2). Shallow earthquakes occur with a remarkable frequency throughout Central America, and several large earthquakes (Mw > 6.5) have taken place along the coast (Figure 3). For example, the 1992 Mw 7.6 Nicaragua earthquake, regarded as a “tsunami earthquake”, occurred at a shallow depth and generated a large tsunami that caused irreversible destruction to several cities in Nicaragua.^15^

Slow earthquakes, including slow slip events (SSEs), have also been observed in the segmented Central American subduction zone, particularly in and around the Nicoya Peninsula of Costa Rica. Global positioning system (GPS) observations between 2002 and 2010 suggest that an SSE occurs beneath the peninsula at an average interval of 21.7 ± 2.6 months, which is equivalent to the release of a 6.6–7.2 Mw earthquake.^20^ The slip area of the SSE that struck in 2007 was divided into shallow and deep parts located updip and downdip, respectively, within the seismogenic zone^9^^,^^21^ (yellow dashed lines in Figures 1 and 2). Moreover, Baba et al.^10^ and Brown et al.^8^ located very-low-frequency earthquakes (VLFEs) and low-frequency earthquakes (LFEs) at shallow and deep depths, respectively, within the Nicoya Peninsula (Figure 1). Over 90% of these VLFEs overlap with the shallow area of the 2007 SSE, while the focal depths of these LFEs are the same depth as the deep portion of the 2007 SSE. Consequently, Baba et al.^10^ analyzed the relationship between slow earthquakes and large earthquakes in the Nicoya Peninsula segment of the Central American subduction zone and proposed that slow earthquakes may occur in the shallower and deeper extents of the rupture regions of large earthquakes. However, the reason for the differential distribution of these slow earthquakes remains enigmatic.

The occurrence of an earthquake in a subduction zone is intimately related to the thermal structure of the subducting slab and the overlying mantle wedge.^22^ Thus, to investigate the relationship between fast and slow earthquakes in the Central American subduction zone, the thermal regime of the subducting Cocos Plate must be accurately known. Recent research has demonstrated that the 3D thermal model constrained by the various observations of surface heat flow shows advantages over the existing 2D thermal models in predicting the along-strike variation in the thermal state of the subducting slab (e.g., Qu et al.^23^^,^^24^). Accordingly, in this study, we estimated the thermal state and water content distribution of the Central American subduction zone based on a 3D thermomechanical model. Then, we compared the modeled results with the observed foci of regular interplate earthquakes, large earthquakes, and slow earthquakes, and we analyzed which main mechanisms could be responsible for generating fast and slow earthquakes beneath Central America.

## Results

### 3D temperature structure of the subducting Cocos Plate

**Figure 4:**
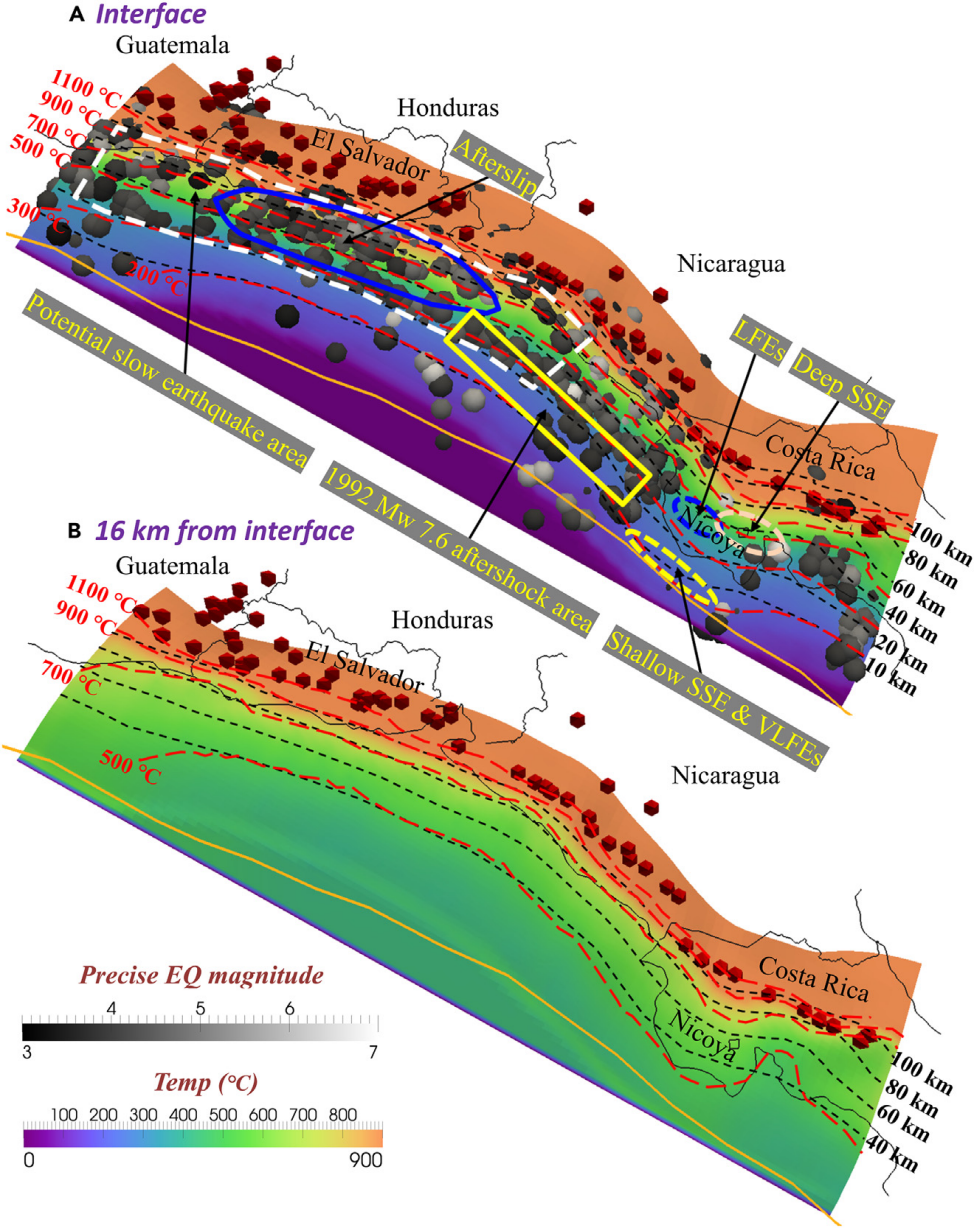
Calculated thermal state of the subducting slab at various depths (measured vertically downward from the slab surface) (A and B) (A) 0 km (the surface); (B) 16 km below the slab surface. Red cones indicate active volcanoes.^3^ The white dashed line indicates the potential slow earthquake area predicted by this study. The blue polygon shows the afterslip area of the 2012 Mw 7.3 tsunami earthquake offshore El Salvador.^19^ The yellow rectangle indicates the aftershock area of the 1992 Mw 7.6 Nicaragua earthquake.^25^ The yellow ellipse with a dashed line shows the distributions of very-low-frequency earthquakes^10^ and the shallow area of the 2007 slow slip event.^9^ The blue ellipse with a dashed line represents the distribution of low-frequency earthquakes.^8^ The light pink ellipse with a dashed line represents the distribution of the deep area of the 2007 slow slip event.^9^

Based on the 3D numerical simulation, we calculated the intraplate temperatures of the subducting Cocos Plate at four isodepth contours (measured downward orthogonal to the slab surface) of 0, 4, 8, and 16 km (Figures 4 and S3). In addition, we relocated the fast and slow earthquakes in the Cartesian coordinate system projected from spherical coordinates; these included all regular earthquakes and slow earthquakes.^6^^,^^7^^,^^19^ Identified slow earthquakes have been located mainly offshore El Salvador^19^ and below the Nicoya Peninsula.^8^^,^^9^^,^^10^ The slab surface temperatures in the source regions of deep SSE and LFEs vary between 300°C and 900°C, whereas shallow SSE and VLFEs occur at lower surface temperatures varying between 200°C and 250°C (Figure 4A). Regular interplate earthquakes occur mainly at depths of 20–80 km at the slab surface with surface temperatures ranging from 300°C to 900°C.

The surface temperature of the subducting Cocos Plate at a depth of 10 km (from Earth’s surface) is mostly approximately 200°C, but the surface temperatures offshore northern Costa Rica are slightly higher than those elsewhere due to the younger age of the slab beneath this region (Figure 4A). The surface temperature of the incoming plate at a depth of 100 km below Nicaragua is approximately 1,100°C. In contrast, the subducted slab beneath El Salvador is warmer, with surface temperatures exceeding 1,100 °C at 100 km depth (Figure 4A). At the Moho (30 km-depth plate interface), temperatures predominantly in the range of 300°C–500°C control the serpentinization occurring within the mantle wedge. Meanwhile, below central Costa Rica, the surface temperature of the subducted slab is lower, decreasing to 300°C –500°C (mantle wedge) at the depth range of 30–60 km, colder than that below El Salvador. The thermal structure of the slab is associated with the geometry of the subducting Cocos Plate. In other words, the variations in the geometry of the slab, such as its dipping angle variations, occur in association with strong temperature variations across strike (Figures 4A and 4B).

According to the calculated results (Figure 4A), we also observed that the subvolcanic slab temperature was as high as >1,100°C on the plate interface. Since the Cocos Plate is warm and thin, the intraslab temperature increases gradually with increasing depth. For example, at the mantle wedge immediately underlying the Moho (30 km) below the central Nicoya Peninsula (Figure 4A), the calculated temperature is ∼400°C, but at greater depth (16 km into the slab), the slab temperature increases to 550°C –600°C (Figure 4B). Such elevated intraslab temperatures indicate the absence of a cold center in the slabs of warm subduction environments like the Central American subduction zone. Furthermore, we noticed a sudden drop in temperature within the incoming plate below central Costa Rica compared to other regions at the same depth (Figure 4B). It indicates that factors apart from slab geometry such as hydrothermal circulation near the trench, slab age, and convergence rate influence the temperature variation in the thermal structure of the slab here.

### 3D water content variation along the strike

**Figure 5:**
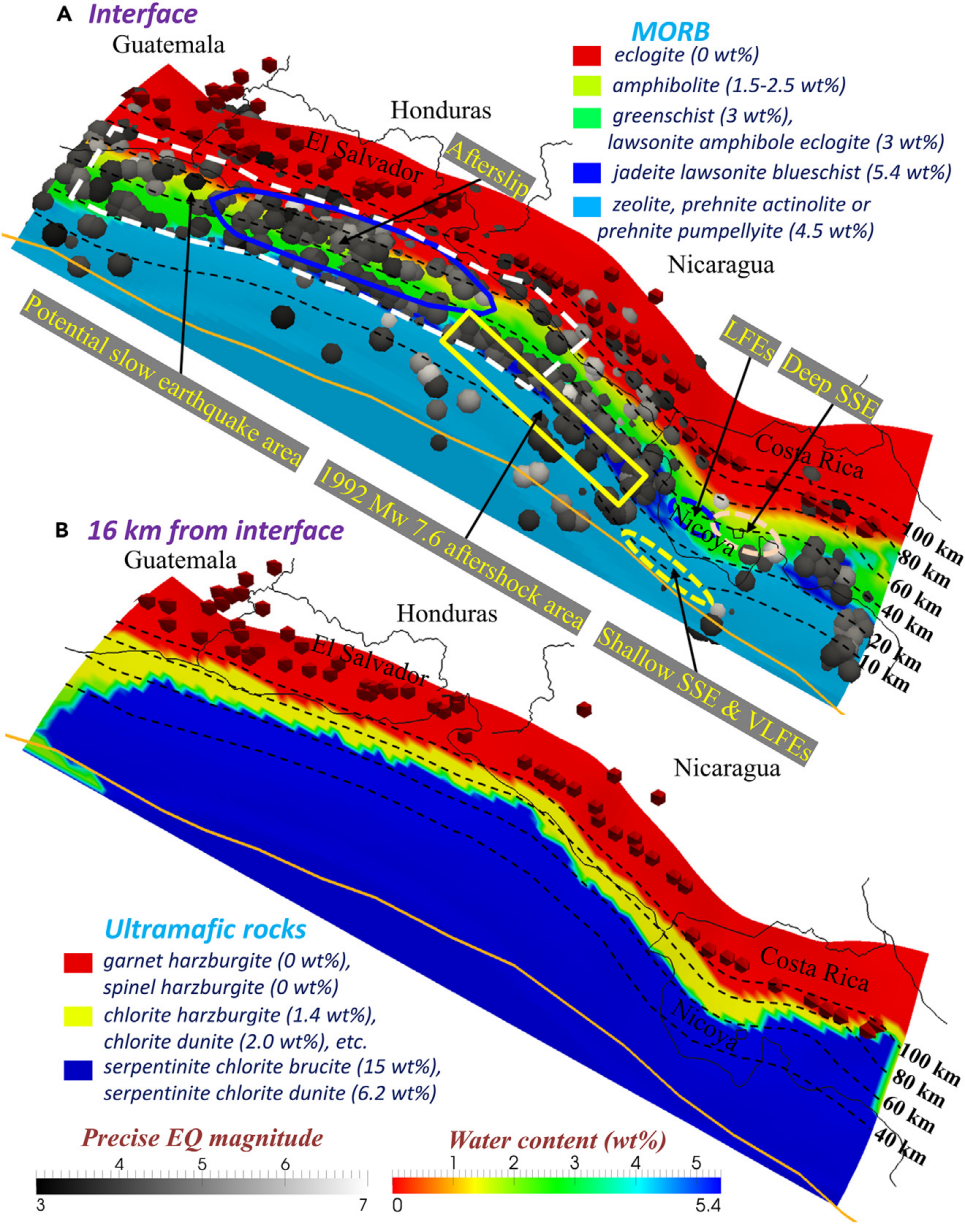
Water content (wt %) of the incoming plate (A and B) (A) 0 km from the interface (MORB); (B) 16 km from the interface (ultramafic rocks). Red cones indicate active volcanoes.^3^

**Figure 6:**
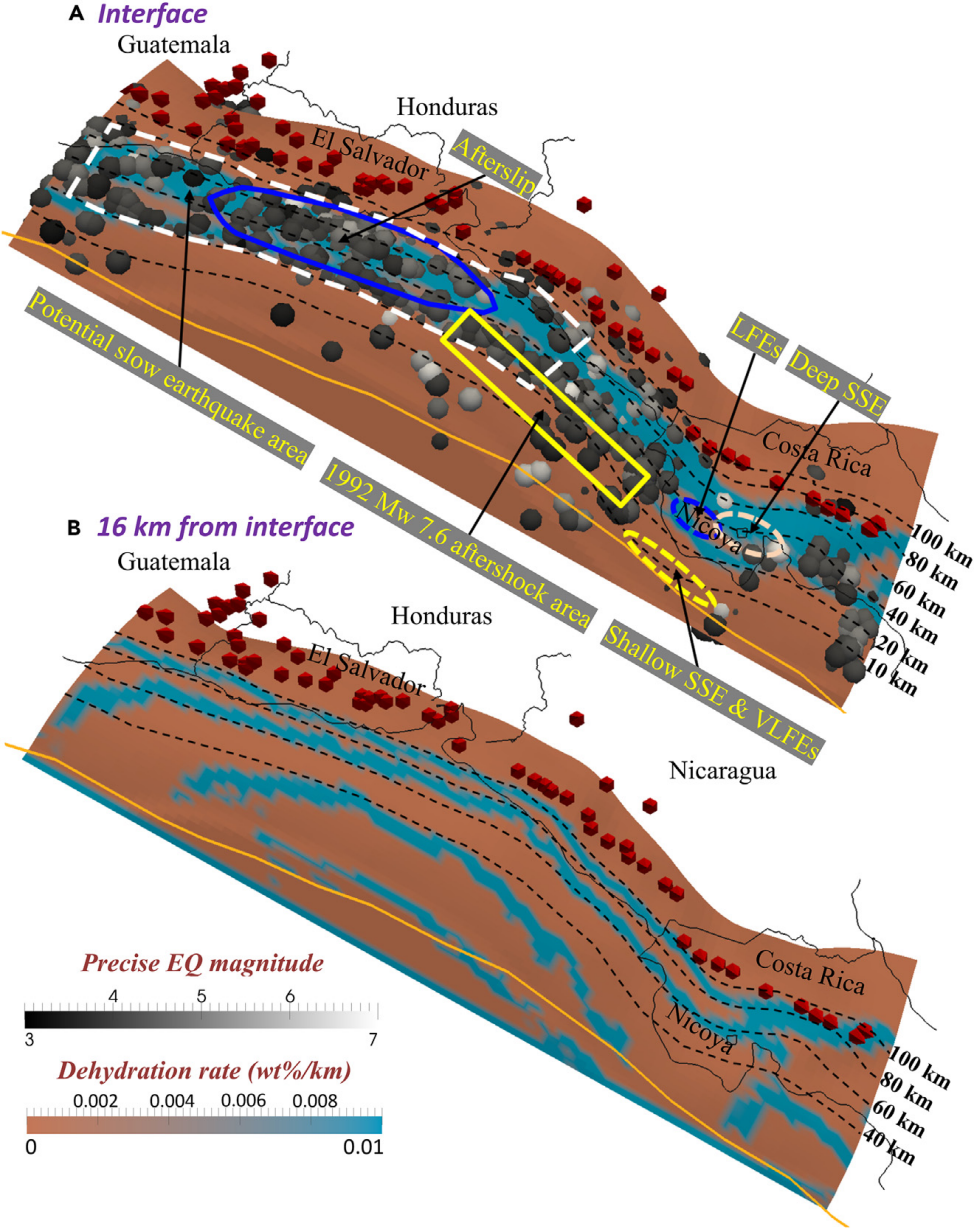
Dehydration (wt %/km) of the incoming plate (A and B) (A) 0 km from the interface; (B) 16 km from the interface. Red cones indicate active volcanoes.^3^

Previous seismic inversion results found that the water content of the subducting Cocos Plate beneath Central America is varying along strike due to differences in crustal thickness, subduction velocity, and dip angle.^26^ To estimate the variation in water content of the slab beneath southern Guatemala to central Costa Rica, we assumed that the rocks were water saturated and calculated the 3D distributions of the slab water content (maximum saturation) (Figure 5) and dehydration (Figure 6) according to the facies diagrams of mid-ocean ridge basalt (MORB)^27^ and ultramafic rocks^28^ in the subducting Cocos Plate. Then, similar to the temperature analysis, we computed the slab water content and dehydration distributions on intraslab isodepth contours of 0, 4, 8, and 16 km below the subducting Cocos Plate interface. The subducting Cocos Plate is assumed to be composed of an upper 7 km thick layer of MORB and a lower layer of ultramafic rocks.^28^ At the shallowest depths (<20 km, Figure 5A), the slab is composed of the zeolite, prehnite-actinolite (PA), and prehnite-pumpellyite (PP) facies with a water content of approximately 4.4 wt %. With increasing depth (20–50 km) beneath the Nicoya Peninsula, the facies of the incoming plate transition into greenschist or lawsonite amphibole eclogite with a water content of 3 wt %. Lawsonite blueschist possesses a relatively high water content of 5.4 wt %, and its spatial distribution varies laterally along the Nicoya Peninsula. Hence, between 0 and 7 km depth, where MORB compositions dominate (Figure 5A), their metamorphism leads to the formation of greenschist (3 wt %) and lawsonite blueschist (5.4 wt %). At depths greater than 7 km in the slab, ultramafic compositions dominate (Figure 5B), and their metamorphism leads to the formation of serpentinite chlorite brucite (15 wt %) and serpentinite chlorite dunite (6.2 wt %).

Based on the above calculations (Figure 6A), we can correlate the slab dehydration transition with the majority of the seismogenic zone location. The dehydration depth of the upper slab layer varies from 15 km to 90 km (Figure 6A), while the dehydration depth of the lower layer varies from 40 km to 100 km (Figure S5A, MORB) or from 60 km to 100 km (Figure 6B, ultramafic layer). Notably, deep SSE, LFEs, and most fast earthquakes occur in the region where the slab dehydration rate is > 0.01 wt %/km. By contrast, although a handful of shallow SSE and VLFEs are located in the updip region of the dewatering zone (e.g., offshore Nicoya Peninsula), only limited slab dehydration and fluid release occur there according to our calculations.

## Discussion

### Comparison with previous thermal structure results

**Figure 7:**
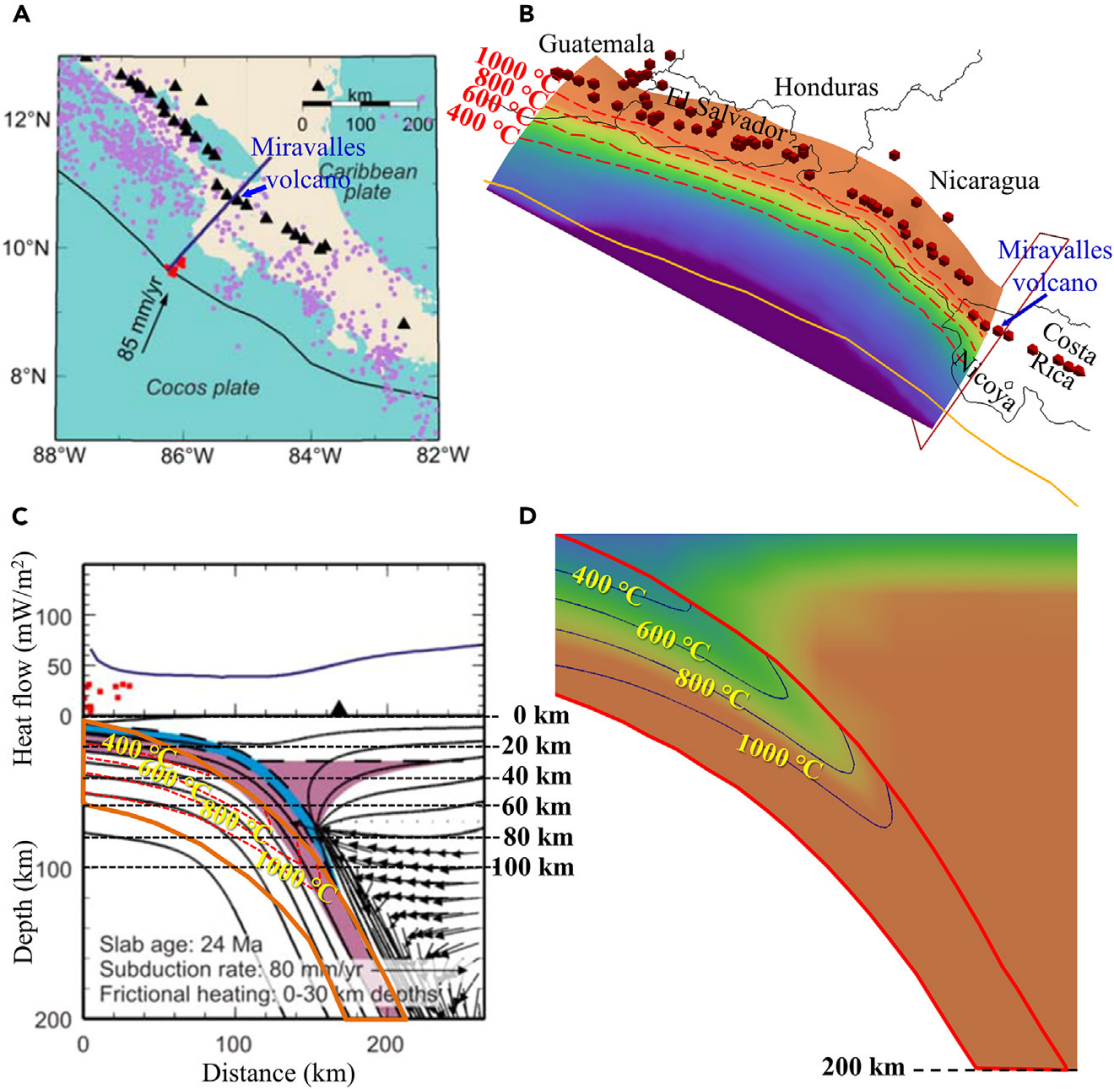
Comparison of model results between this study and previous study (A) The profile crossing Miravalles volcano is used in thermal modeling.^29^ (B) The same profile is from the 3D model in this study. Red cones indicate active volcanoes.^3^ (C) Slab geometry difference and calculated thermal state difference between Wada and Wang^29^ and this study. The black curves and symbols are from Wada and Wang,^29^ while the orange curves (top and bottom surfaces of the slab) and red dashed curves (intraslab thermal contours) are the results of this study. (D) Cross-sectional thermal structure along the same profile in this study.

Multiple 2D thermal models have been developed and applied to the Central American subduction zone beneath Nicaragua and Costa Rica, and the slab thermal structure has been estimated along several profiles (e.g., Peacock et al. and Wada et al.^22^^,^^29^). Wada and Wang^29^ calculated the temperature variation along the profile crossing the Miravalles volcano in Costa Rica (Figures 7A and 7C) based on the slab-mantle (or interplate) decoupling model. The constraints of the model can severely affect the calculated subduction thermal regime. In our 3D model, the thermal regime is constrained by various observations of surface heat fluxes, including Curie point depth estimations^30^ and the prescribed plate coupling, which we believe is better at predicting the thermal state of the subducting slab. Qu et al.^23^ suggested medium-high heat flows (50–140 mW/m^2^) beneath northeast Japan and the Cascadia forearc, indicating that the forearc is not as cold as described by the slab-mantle (or interplate) decoupling model.^29^ In Central America, we also found high heat flows (>80 mW/m^2^) at the trenchward forearc (e.g., offshore Costa Rica) (Figures 2 and S1), which may indicate that the subducting slab surface temperature is warmer than previously thought. Therefore, we adopted our model to reevaluate the temperature range of the cold mantle wedge (<500°C) and constrained the bottom of the cold nose to depths of 40–60 km (Figures 7B and 7D).

The thermal structure of the subducting slab depends on many parameters, including the age of the subducting lithosphere, convergence rate, geometry, rate of shear heating, and so on.^31^ Maunder et al.^32^ found that the slab age and speed have the most important effects on the slab surface temperature. Following the isoviscous mantle-wedge rheology and the olivine mantle-wedge rheology, Peacock et al.^22^ predicted slab surface temperatures beneath Nicaragua and Costa Rica of approximately ∼620°C and ∼800 °C at 3 GPa (100 km depth), respectively. Correspondingly, we calculated the temperature variations along the same three profiles as in Peacock et al.^22^ passing through volcanic areas in Nicaragua, northern Costa Rica, and central Costa Rica (Figure S2A). Our thermal results reveal that at the same depth (100 km), the slab surface temperatures beneath Nicaragua and northern Costa Rica are ∼1,000°C (Figures S2B and S2C), while the slab surface temperature beneath central Costa Rica rises to 1,200°C (Figure S2D). Compared with previous 2D models, our model is jointly constrained by a variety of additional morphological controlling factors such as the slab curvature, which is relatively sensitive to modest variations in the age of the subducting lithosphere and convergence rate (e.g.,^33^^,^^34^^,^^35^). Overall, we find that the surface temperature of the subducting Cocos Plate beneath Central America is much warmer than previous studies have suggested, which is consistent with the surface heat flow and seismicity observations.

Conductive lithospheric cooling models have predicted heat flows of 95–120 mW/m^2^ for 18–24 Myr seafloor.^36^ However, Fisher et al.^37^ observed particularly low seafloor heat flows (20–40 mW/m^2^) near the Middle American Trench and inferred the occurrence of vigorous hydrothermal circulation in the shallow basement that extracts heat. Vigorous fluid circulation can affect subduction zone temperatures and thus significantly change the thermal structure of subducting plate.^38^^,^^39^^,^^40^ Hass and Harris^41^ incorporated hydrothermal circulation into their thermal models, and their results found that the incoming plate beneath central Costa Rica is warmer near the trench and colder away from the trench. From our results, since the changes in temperature of the slab beneath central Costa Rica shown in Figure 4 are consistent with their findings, we thus infer that hydrothermal circulation also contributes to the variation in thermal structure of the subducting plate beneath Central America along strike.

### Distribution of fast-slow earthquakes in the central American subduction zone

We further investigated the ambient conditions required for fast and slow earthquakes to occur at various depths and sought to discover the interconnection among them. We observed that, except for a few earthquakes scattered at shallow depths, most of these earthquakes are distributed at absolute depths of 20–80 km, which is related to the dewatering zone of >0.01 wt %/km (Figure 6). Fluids are typically generated from within the slab by multistage metamorphic reactions requiring high-temperature and high-pressure conditions associated with depth,^42^^,^^43^ but our results regarding the water content variation suggest that the upper depth limit for the dewatering zone on the upper slab surface is 20 km, which may be attributable mainly to the fact that the shallow slab is characterized by such high temperatures. Moreover, the widely accepted mechanism for the generation of intermediate-depth (50–300 km) earthquakes is dehydration embrittlement from the breakdown of hydrous minerals within the subducting slab.^28^^,^^44^ We consider that dehydration embrittlement could also have implications for shallow (20–50 km) seismicity in Central America, which is consistent with the observed dewatering zone of the slab surface. Therefore, we propose that warm-plate-environment-controlled dehydration may be the main driving mechanism for fast and slow earthquakes along the Central American subduction zone.

Previous studies have noted that the generation mechanisms of shallow and deep SSEs may be similar.^21^ At shallow depths, fluids can be released by the compaction of subducted sediments atop the slab.^43^^,^^45^ Rüpke et al.^46^ modeled the fluid release beneath Nicaragua and Costa Rica, and their results showed that the sediments released ∼75% of the chemically bound water during shallow (depth <50 km) dewatering. Our results suggest that deep SSE and LFEs (30–50 km) occur on the slab surface in the dewatering zone, while shallow SSE and VLFEs (6–10 km) occur elsewhere. Outerbridge et al.^9^ suggested that the occurrence of SSEs at a shallow depth is attributed to the updip transition from stick-slip to stable sliding. However, this transition has difficulty occurring at such shallow depths considering the change in interplate friction conditions. Thus, we stipulate that fluids derived from the compaction of subducted sediments promote the occurrence of shallow SSEs. Overall, beneath the Nicoya Peninsula, fluids were released in different ways and at different depths, ultimately leading to an increase in the pore fluid pressure, which promoted SSEs on the megathrusts atop a young and hot segmented slab beneath Costa Rica with the seafloor age of 16–25 Ma. Furthermore, partially released fluids are conjectured to be stored in the crust beneath the Nicoya Peninsula.^47^ Stored fluids accumulated over a long time and triggered ruptures of earthquakes. This may explain why the Nicoya Peninsula lacks regular interplate earthquakes but experienced several large earthquakes (Figure 3, yellow stars and white stars).

### Potential slow earthquakes in the Central American subduction zone

When the subducting plate interface is in a fluid-rich environment characterized by high pore pressures, the fluid overpressure reduces the effective normal stress and weakens the plate interface, thereby facilitating brittle failure and ultimately causing the occurrence of SSEs.^48^^,^^49^ Beneath Central America, the presence of the observed dewatering zone on the plate interface from Guatemala to Costa Rica provides similar support for the generation of slow earthquakes. Previous studies located the aftershock area of the 1992 Mw 7.6 Nicaragua earthquake and suggested that it was a slow tsunami earthquake associated with subducted sediments^18^^,^^25^ (Figure 4A). Furthermore, Geirsson et al.^19^ estimated the seismic deformation from the 2012 Mw 7.3 tsunami earthquake offshore El Salvador and found significant afterslip, causing the event to manifest as exhibiting slow slip over a large region (Figure 4A). The aftershock area and afterslip area indicate that the plate contact offshore from El Salvador to Nicaragua is weakly coupled and unstable, similar to that of Costa Rica. Consequently, we suggest that the northern-central part of the Central American subduction zone has the potential for slow earthquakes to occur, particularly in the Guatemala-Nicaragua segment of the heterogeneous megathrust (Figure 4A, surrounded by a dashed white line).

Considering that slow earthquakes require long-term observations over several years to decades,^20^ researchers deployed multiple observational networks in the northern-central part of Central America that can provide continuous data.^50^^,^^51^^,^^52^ However, compared to the dense instrumental network located in the Nicoya Peninsula, the lack of near-trench geodetic monitoring in the Guatemala-Nicaragua segment makes detecting slow earthquakes extremely challenging.^53^ Moreover, the influence from regular interplate earthquakes (Figure 4A, M > 3), the noise in GPS data,^20^ and instrumental responses^10^ also make it difficult to distinguish SSEs and LFEs from the data. In this case, previous studies have been more focused on using models to predict the probability of slow earthquake occurrence. For example, McLellan and Audet^54^ predicted the occurrence probability of SSEs in Central America and showed that their occurrence (durations of days to a few weeks) is the highest in northern Central America.

Furthermore, as the subducting plate changes curvature in three dimensions, fluid pore pressure can hence be affected and follow areas where the surrounding stress (including resultant brittle failures) allows them to flow.^55^^,^^56^ For instance, some 2D and 3D flexural bending studies have found that the stress and deformation of the subducting slab can exert influence on the temperature of the slab and the fluid transport.^57^^,^^58^^,^^59^ In addition, the potential impact from the overriding plate and its thermomechanical controls cannot be ruled out in terms of stress coupling. If the stress and strain evolution of the slab-mantle system is further investigated in the modeling, the composite of subduction regime could be obtained at a higher accuracy, and this work will be left for future studies.

### Conclusions

According to 3D thermomechanical modeling of the Cocos Plate subducting beneath Central America from Guatemala to Costa Rica, the following conclusions are drawn.

(1) The surface temperature of the incoming plate beneath Central America is warmer than previously thought, and the cold mantle wedge (<500°C) below the Central American subduction zone is constrained at depths of 40–60 km.
(2) Warm-plate-environment-controlled slab dehydration appears to be the mechanism responsible for the recurrence of fast and slow earthquakes along the heterogeneous Central American megathrusts at depths of 20–80 km.
(3) Fluids derived from subducted sediments at shallow depths and slab metamorphism at greater depths below the Nicoya Peninsula and the Guatemala-Nicaragua segment of megathrusts result in the increased pore fluid pressure and promote the occurrence of slow earthquakes over a broad depth range.

### Limitations of the study

In this study, we found that the surface temperature of the Cocos Plate is warmer than previously estimated. As the plate subducted, warm-plate-environment-controlled slab dehydration led to the release of more fluids and drove the occurrence of fast-slow earthquakes in the Central American subduction zone. The limitations of the current study are mainly attributed to the modeling settings; for example, the aquifers are not taken into account, which may result in low surface heat flow at the Nicoya Peninsula. This causes some uncertainty in the calculated temperature of the shallow part (depth <10 km) of the subducted plate. On the other hand, there is some uncertainty in the calculation of plate water content variation. We utilized a water content of 15 wt % for the subducting oceanic plate because harzburgite is the dominant rock type in the uppermost oceanic mantle, which is largely serpentine at lower temperatures.^28^ However, the estimates remain uncertain because serpentinites at low temperatures (50°C –300°C) are suggested to be mainly composed of lizardite and magnetite with brucite, but chlorite is not common.^60^

## STAR★Methods

### Key resources table

**Table:** 

REAGENT or RESOURCE	SOURCE	IDENTIFIER
**Deposited data**		

The 3-D thermal model results in this study	Mendeley Data	https://data.mendeley.com/datasets/vdkmh9cz5r
Stag3D code	Tackley et al.^62^	https://doi.org/10.1016/B978-008044046-0.50372-9
Incorporated Research Institutions for Seismology	Trabant et al.^6^	https://doi.org/10.1785/0220120032
Slab 2	Hayes et al.^5^	https://doi.org/10.1126/science.aat4723
ETOPO Global Relief Model	Smith et al.^4^	https://doi.org/10.1126/science.277.5334.1956

**Software and algorithms**		

Generic Mapping Tools	Wessel et al.^61^	https://doi.org/10.1029/98EO00426
Paraview	Kitware Inc.	https://www.paraview.org/license/

### Resource availability

#### Lead contact

Further information and requests for resources should be directed to the lead contact, Yingfeng Ji, yingfengji@itpcas.ac.cn.

#### Materials availability

This study did not generate new unique reagents.

### Experimental model and subject details

Our study does not use experimental models.

### Method detail

The developed models were adopted from thermomechanical codes (Stag3d^62^) utilizing the finite difference method (FDM). We performed a 3-D, time-evolving numerical simulation of the slab of the Cocos Plate in the Central American subduction zone ranging from southern Guatemala to central Costa Rica. In our study, we applied an anelastic liquid approximation and the equations corresponding to the conservation of mass, momentum, and energy and ensured that each of these conditions was satisfied in the model calculation.^63^^,^^64^ The model is 1000 km wide by 500 km long by 400 km deep (Figure 3). The grid number is 80 × 80 × 100. Based on this grid, the number of iterations in our calculation exceeds one thousand, and the total calculation time in the Sugon supercomputing platform takes one week. Moreover, we tested the resolution and found that the temperature variance was <1%, with a maximum temperature variance <1.9% between meshes of 80 × 80 × 100 and 96 × 96 × 100. We prescribe the subduction duration to be at least ≥20 Myr to ensure that the model reaches a steady thermal state with a temperature variation <10°C over time with a lapse time of ≥5 Myr.

The crust of the subducting Cocos Plate was generated at two different spreading centers (the East Pacific Rise and the Cocos-Nazca Spreading Center) in the Central American subduction zone.^65^^,^^66^ The crust formed at the East Pacific Rise moved northeastward with respect to Central America and subducted beneath Guatemala to northern Costa Rica, whereas the crust formed at the Cocos-Nazca Spreading Center moved northwestward with respect to Central America and subducted beneath central and southern Costa Rica.^12^ Due to the difference in the direction of movement, the tectonic boundary defined by the magnetic data indicates that they are orthogonal to the Central American Trench off the central Nicoya Peninsula.^67^ The seafloor age of the former crust near the trench is ∼24 Ma, and that of the latter crust near the trench is 16∼24 Ma (Figure 2). To correctly simulate the age distribution of the incoming plate, we interpolated the age of the oceanic lithosphere along the trench estimated at the trenchward model boundary according to EarthByte.^17^ Based on the known seafloor age near the trench, the age at each grid node inside the kinematically prescribed slab domain is determined by linear interpolation in combination with the subduction direction and the slab geometry. Since more than 20 Myr of the subduction time is required to ensure that the model reaches a steady thermal state, the starting point of our model calculations begins at least from 44 Ma. These plate ages were crucial to constructing the thermal structure of the subducting Cocos Plate because the younger the age the hotter the plate. The variation in plate age could significantly change the slab dehydration temperature at depth and determine whether the subduction system was a hot endmember or cold endmember system. Thus, the intraslab dehydration fronts could be correctly estimated in accordance with the variation along strike. If the seafloor ages with errors smaller than 1 Ma,^17^ it induces a small temperature variation of <10 °C at a depth of 30 km in our model that follows the Global Depth and Heat (GDH1) model.^68^^,^^69^ Furthermore, aquifers are not considered in this model, and the downgoing lithosphere geometry was kinematically prescribed without slab surface deformation during time step, and the density and viscosity of each layer (e.g., upper crust, lower crust, subducted slab, mantle lithosphere, and asthenosphere, for continent and ocean, etc.) remain constant (e.g., Tables in Supplemental Information). Due to the influence of variation in density or stress on the thermal structure of the slab, it is not necessary to include more complex relationships such as Boussinesq density, power-law water dependent rheology, yield criteria, and so on. In turn, if we consider this long-term influence (e.g., density variation by 200 kg/m^3^), it will lead to a <10% uncertainty in the main conclusions, which is sufficient for our study.

The geometry of the subducted slab is well constrained by Slab2.0,^5^ and the intraslab subduction velocities follow the MORVEL dataset 1. The oceanic lithosphere thickness was estimated by the plate age,^70^ and the temperature boundary condition was controlled according to the cooling of the oceanic lithosphere.^69^ The bottom of the slab and the vertical plane were prescribed as adiabatic (dDT = 0) and permeable (dV≠0), and the top surface was set to be a fixed temperature (0°C). Since we did not focus on the long-term tectonic geomorphology in this study, the top surface was prescribed as rigid, thus, we did not need to resolve the top surface deformation processes and vertical to lateral force equilibrium. The subducting oceanic lithosphere is composed of a 7 km thick mid-ocean ridge basalt (MORB) layer at the top and a lower layer composed of ultramafic rocks.^28^ Based on Omori et al.^27^ (MORB) and Hacker et al.*,*^28^ we established a P-T-wt%-facies database with a P-T grid interval of 0.04 GPa (1.2 km) and a temperature interval of 5°C. The temperature and pressure at each P-T grid point were calculated from the model and the preliminary reference Earth model (PREM). Through interpolation, we derived the intraslab water content distribution (wt %) and dehydration gradient (wt %/km) at different depths.

### Quantification and statistical analysis

Observations of surface heat flow^16^ and published Curie point depths^30^ were employed to constrain the model thermal regime (Figure S1). During the calculation, we used the least square method to compare the calculation with the observed heat flow values, then linearly interpolated the parameters (e.g., plate depth, subduction velocity, and plate age) and ran the program again until we obtained the optimal values as the results. Synthetic modeling suggests that the largest error in the Curie depths estimated using the linearized centroid method is within 35%, and the uncertainty in the surface heat flow is expected to be <20 mW/m^2^ due to the selected fractal exponent and wavenumber bands for the linear regressions and observed surface heat fluxes in zones of plate convergence.^30^ If the heat flow error is maximized (20 mW/m^2^), the uncertainty in temperature is approximately 20∼30%. According to the fixed parameters (Supplemental Information) we chose, the uncertainty in the thermal results upon reaching a steady state is < 10°C, which remains within an acceptable range. Thus, we performed sensitivity tests to investigate the robustness of our modeling results by varying the mantle viscosity and density, and the benchmark model results are presented in terms of their deviations from the reference models. The tests show that the mantle density variations (±50 kg/m^3^) induce small temperature variations of <10°C at different depths.

## Data Availability

• The data produced in this study can be downloaded (publicly accessible) via Mendeley Data: https://data.mendeley.com/datasets/vdkmh9cz5r. • This paper does not report original code. • For any additional information required to reanalyze the data reported in this paper, please contact the lead author.
