# Supplementary material for: Variation in the thermal and dehydration regime below Central America: Insights for the seismogenic plate interface
Source: iScience. 2023 Sep 17;26(10):107936. doi: 10.1016/j.isci.2023.107936 (PMC10561039; doi:10.1016/j.isci.2023.107936)
Supplement: Document S1. Figures S1–S5 and Tables S1 and S2 [file mmc1.pdf]

iScience, Volume *26*

Supplemental information

Variation in the thermal and dehydration regime below Central America: Insights for the seismogenic plate interface

Rui Qu, Yingfeng Ji, Lijun Liu, Weiling Zhu, Ye Zhu, Chaodi Xie, Shoichi Yoshioka, Haris Faheem, and Lin Ding

Table S1. Main model parameters

	Model Parameters	Value	Units
ρ_0	Standard density	3300[a]	$kg\cdot m^{-3}$
α_0	Standard thermal expansion	3×10^{-5}[b]	K^{-1}
T_0	Standard temperature	1600	K
k_0	Standard thermal conductivity	2.9[c]	$W\cdot m^{-1}\cdot K^{-1}$
Hr	Radioactive heat generation rate in the mantle	2.245×10^{-13}[a]	$W\cdot m^{-3}$
C_{p_0}	Standard specific heat at constant pressure	1200[a]	$J\cdot kg^{-1}\cdot K^{-1}$
κ_0	Standard thermal diffusivity	7.6×10^{-7}[d]	$m^2\cdot s^{-1}$
η_0	Standard viscosity	1×10^{20}[c]	$Pa\cdot s$
v	Subduction velocity	6.8–8.0[e]	$cm\cdot y^{-1}$

		Diffusion creep[f]	Dislocation creep[f]
n_0	Stress exponent	1.0	3.5
A_0	Pre-exponential factor	1.0	9.0×10^{-20}
C_{OH}	OH concentration ($H/10^6$ Si)	1000	1000
r	C_{OH} exponent	1.0	1.2
E_0	Activation energy (kJ/mol)	335	480
V_0	Activation volume (m^3/mol)		
	Upper mantle	4.0×10^{-6}	11.0×10^{-6}
	Lower mantle	1.5×10^{-6}	-
d	Grain size (µm)		
	Upper mantle	10000	-
	Lower mantle	40000	-

[a] Wang *et al.* [1] [b] Iwamori [2] [c] Christensen [3] [d] Yoshioka and Murakami [4]
[e] DeMets *et al.* [5] [f] Hirth and Kohlstedt [6]

Table S2. Parameters for all domains

Domains	Upper crust	Lower crust	Slab	Mantle	Accretionary prism
Density (kg/m^3)	2600	2900	3300	3300	2600
Viscosity (Pa·s)	1×10^{-20}	1×10^{-20}	1×10^{-20}	1×10^{-20}	1×10^{-20}
Radioactive heat (W/m^3)	7.3×10^{-10}	1.4×10^{-10}	2.245×10^{-13}	2.245×10^{-13}	7.3×10^{-10}
for magnetic layers:					
oceanic	1.37×10^{-6}				
continental	2.0×10^{-6}				
Thermal conductivity (W/m·K)	2.5	2.5	2.5	2.5	1.4
for magnetic layers:					
oceanic	2.0				
continental	2.5				

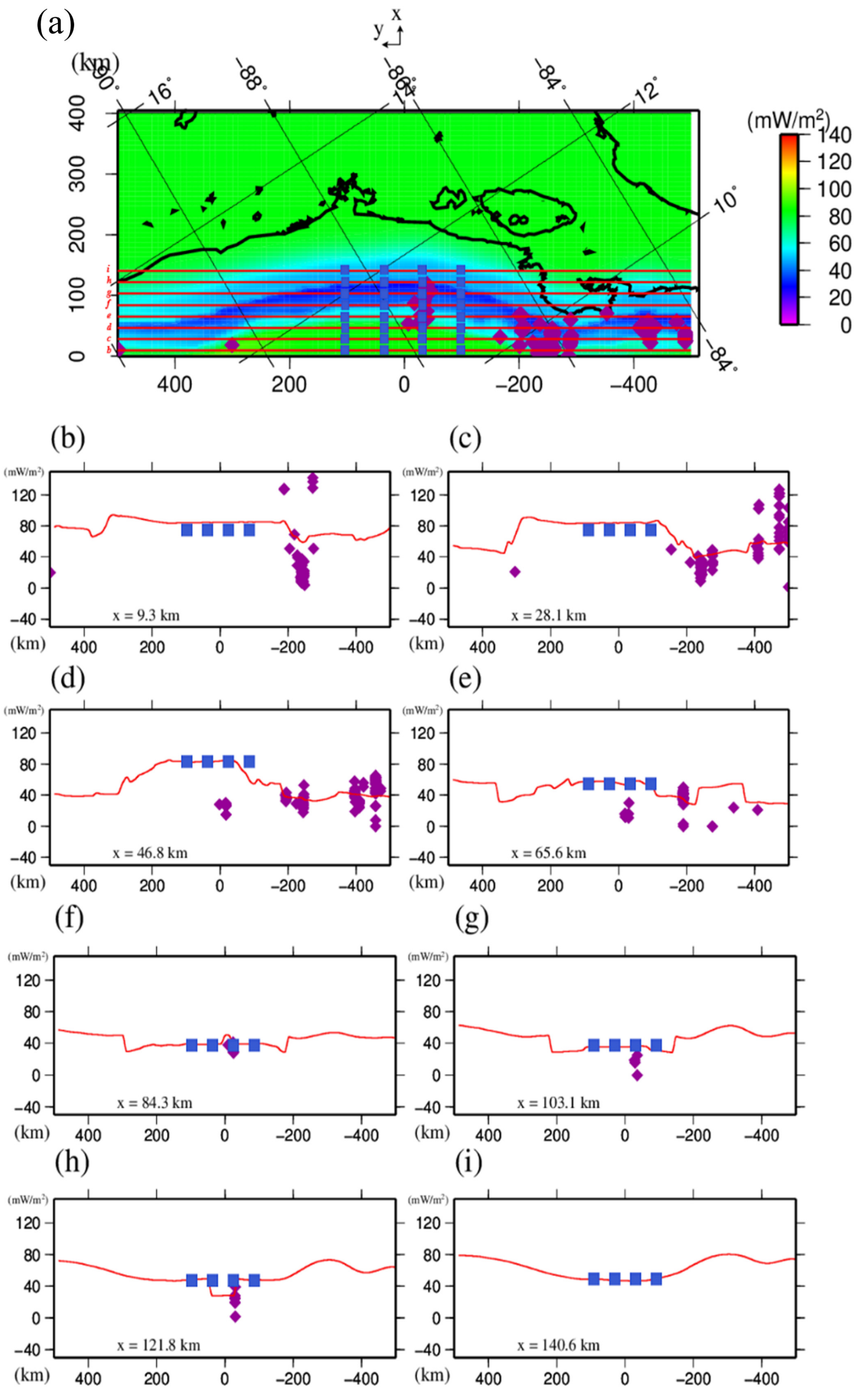

◆ Global heat flow database (Pollack *et al.* [7])

■ Heat flow from Curie depth point estimation (Li *et al.* [8])

Figure S1. Spatial distribution of the surface heat flow calculated in this study. The observed and calculated surface heat flows are compared along trench-parallel profiles "b" to "i" in panels (b) to (i). (b) Observed and calculated heat flows along profile b (x = 9.3 km) in (a). Purple diamonds and blue squares denote heat flow data from the global heat flow database [7] and heat flow from Curie point depth estimates [8], respectively, within a width of 9.4 km along the profile. The red curve indicates the calculated heat flow along the profile. (c) Along profile c (x = 28.1 km). (d) Along profile d (x = 46.8 km). (e) Along profile e (x = 65.6 km). (f) Along profile f (x = 84.3 km). (g) Along profile g (x = 103.1 km). (h) Along profile h (x = 121.8 km). (i) Along profile i (x = 140.6 km).

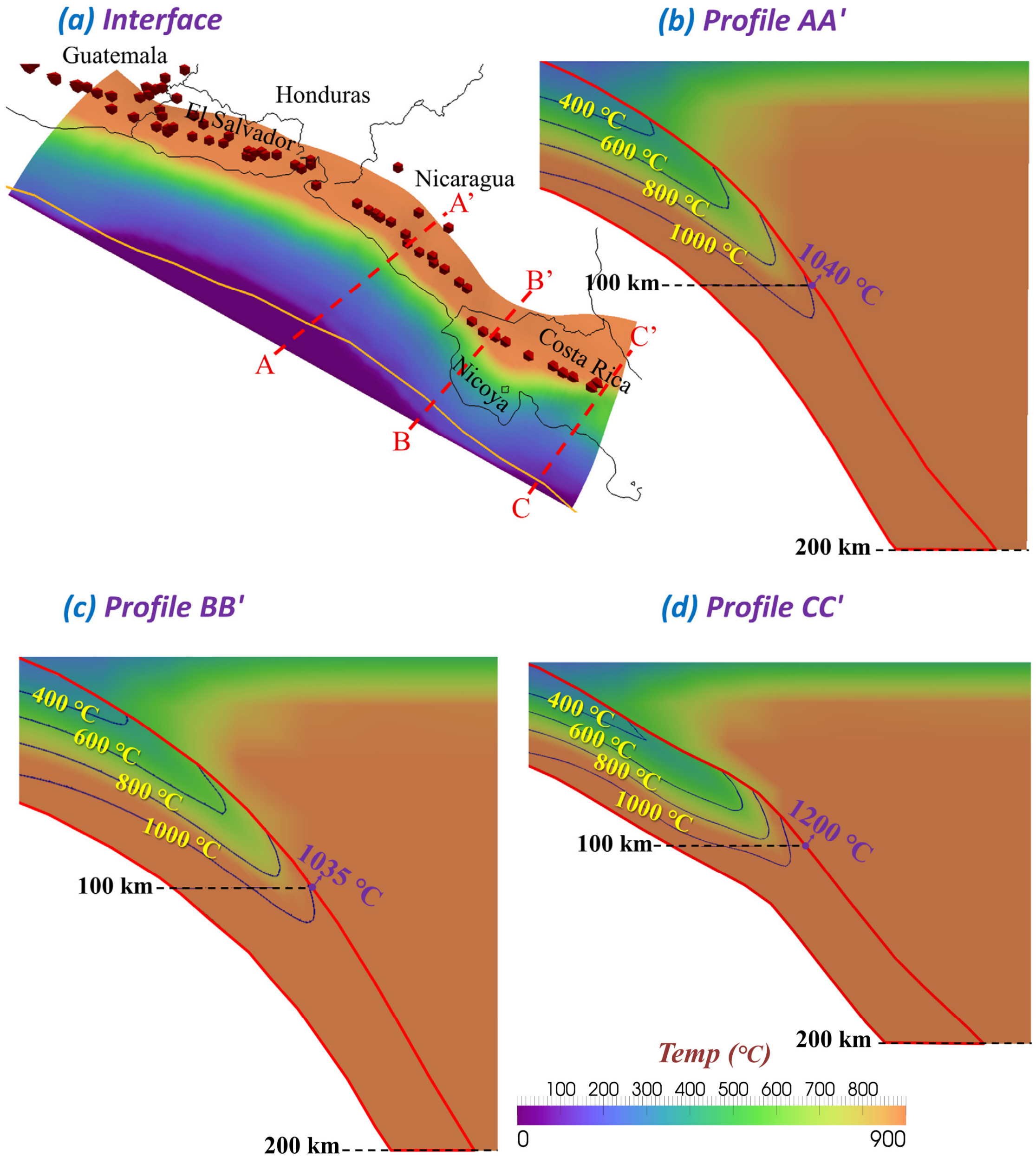

Figure S2. Comparison of the modeling results between this study and Peacock *et al.* [9]**.** (a) Interplate temperature calculated in this study. The profiles AA' (b), BB' (c), and CC' (d) are used to plot the cross-sectional thermal structure which is compared with Peacock *et al.* [9]. Red cones indicate active volcanoes [10].

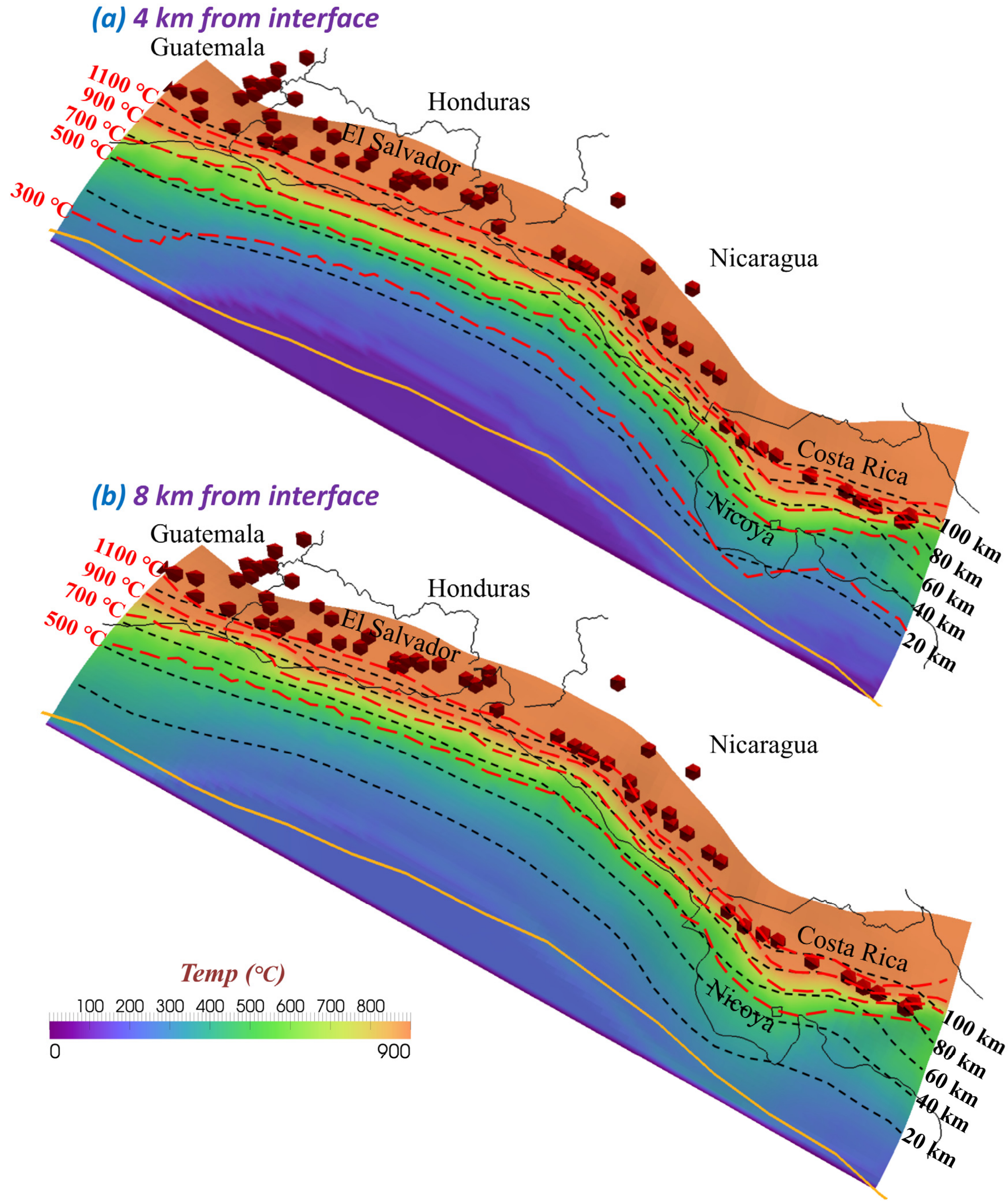

Figure S3. Calculated thermal state of the subducting slab at various depths (measured vertically downward from the slab surface). (a) 4 km below the slab upper surface; (b) 8 km below the slab upper surface. Red cones indicate active volcanoes [10].

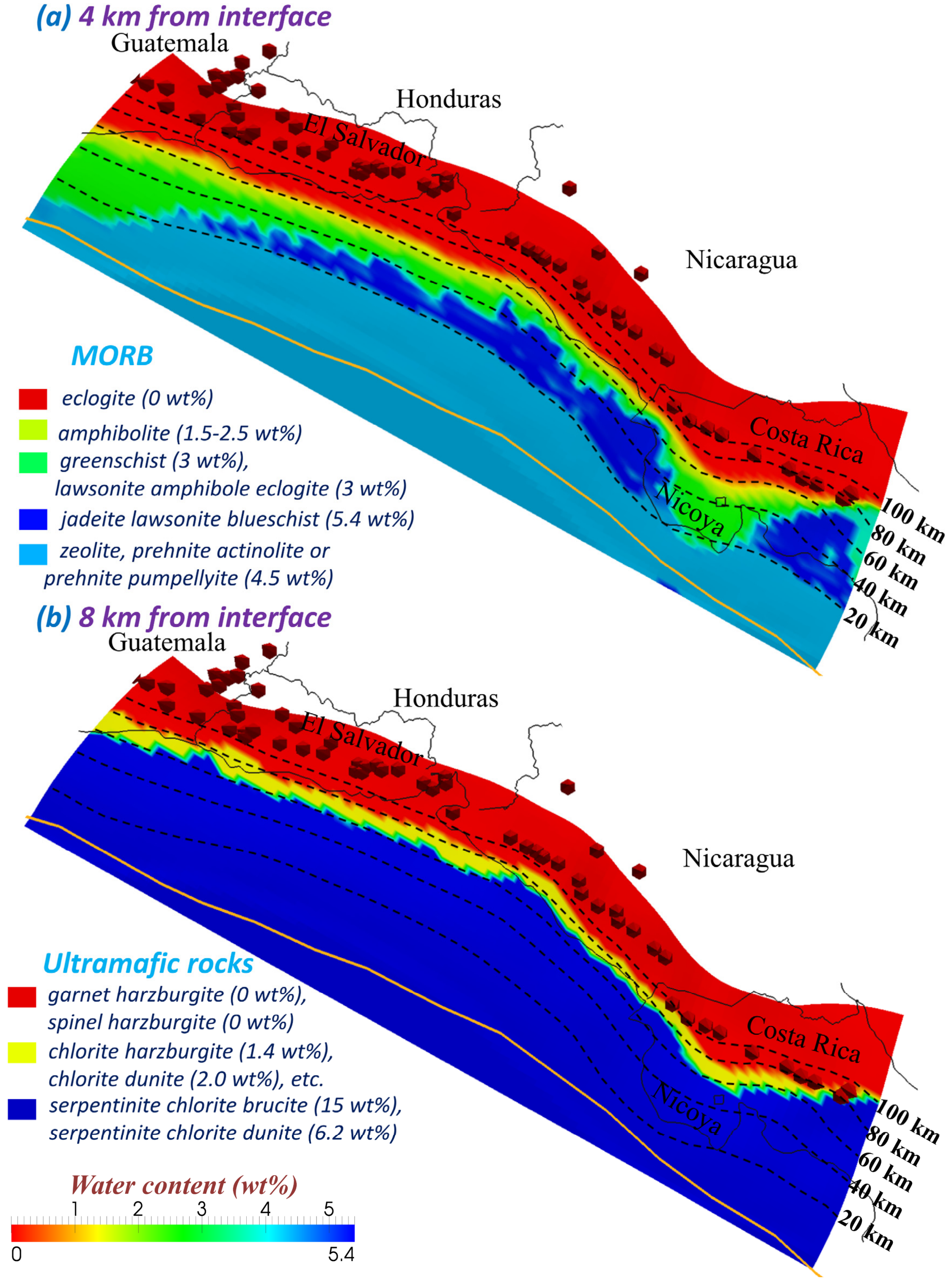

Figure S4. Water content (wt%) distribution of the subducting slab in this study. (a) 4 km below the slab upper surface; (b) 8 km below the slab upper surface. Red cones indicate active volcanoes [10].

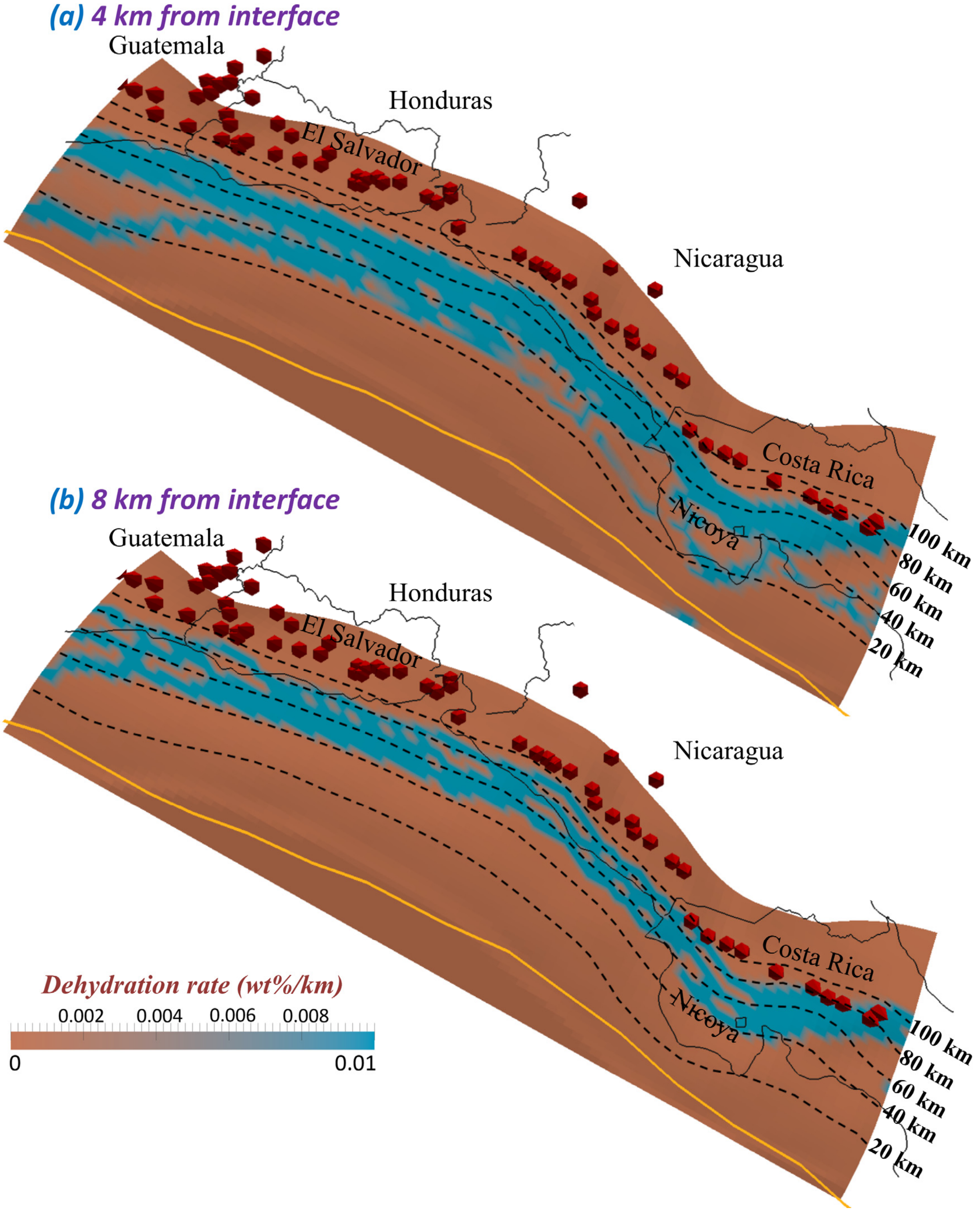

Figure S5. Slab dehydration (wt%/km) distribution of the subducting slab in this study. (a) 4 km below the slab upper surface; (b) 8 km below the slab upper surface. Red cones indicate active volcanoes [10].

References

1. Wang, K., Hyndman, R.D., and Yamano, M. (1995). Thermal regime of the Southwest Japan subduction zone: effects of age history of the subducting plate. Tectonophysics *248*, 53-69.
2. Iwamori, H. (1997). Heat sources and melting in subduction zones. Journal of Geophysical Research: Solid Earth *102*, 14803-14820.
3. Christensen, U.R. (1996). The influence of trench migration on slab penetration into the lower mantle. Earth and Planetary Science Letters *140*, 27-39.
4. Yoshioka, S., and Murakami, K. (2007). Temperature distribution of the upper surface of the subducted Philippine Sea Plate along the Nankai Trough, southwest Japan, from a three-dimensional subduction model: relation to large interplate and low-frequency earthquakes. Geophysical Journal International *171*, 302-315.
5. DeMets, C., Gordon, R.G., and Argus, D.F. (2010). Geologically current plate motions. Geophysical Journal International *181*, 1-80.
6. Hirth, G., and Kohlstedf, D. (2003). Rheology of the upper mantle and the mantle wedge: A view from the experimentalists. Geophysical monograph-american geophysical union *138*, 83-106.
7. Pollack, H.N., Hurter, S.J., and Johnson, J.R. (1993). Heat flow from the Earth's interior: analysis of the global data set. Reviews of Geophysics *31*, 267-280.
8. Li, C.F., Lu, Y., and Wang, J. (2017). A global reference model of Curie-point depths based on EMAG2. Scientific reports *7*, 1-9.
9. Peacock, S.M., van Keken, P.E., Holloway, S.D., Hacker, B.R., Abers, G.A., and Fergason, R.L. (2005). Thermal structure of the Costa Rica-Nicaragua subduction zone. Physics of the Earth and Planetary Interiors *149*, 187-200.
10. Siebert, L., Simkin, T., and Kimberly, P. (2010). Volcanoes of the World.
